# Retraction: Epithelial-Mesenchymal Transition Markers and HER3 Expression Are Predictors of Elisidepsin Treatment Response in Breast and Pancreatic Cancer Cell Lines

**DOI:** 10.1371/journal.pone.0298557

**Published:** 2024-02-05

**Authors:** The PLOS ONE Editors

After this article [1] was published, concerns were raised about results reported in Figs 2–5.

Specifically:

- The Vimentin MDA-MB-468 panel in Fig 2A appears similar to the Vimentin MCF-7 panel in Fig 2A.
- The Slug MDA-MB-468 panel in Fig 2A appears similar to the Twist AsPC-1 panel in Fig 3A.
- The Snail SKBR3 panel in Fig 2A appears similar to the Snail CFPAC panel in Fig 3A.
- The central region of the β-catenin B1474 panel in Fig 2C appears similar to the MCF-7 panel in Fig 4A.
- The E-cadherin PANC-1 panel in Fig 3A appears similar to the E0cadherin MiaPaCa-2 panel in Fig 3A.
- The β-actin panel in Fig 3B appears similar to the right β-actin panel in Fig 4B.
- The Vimentin AsPC-1 panel in Fig 3C appears similar to the β-catenin BxPC-3 panel in Fig 3C when colour is adjusted.
- The upper central region of the E-cadherin BxPC-3 panel in Fig 3C appears similar to the BT474 panel in Fig 4A.
- The HPAC Snail panel in Fig 5A appears similar to the HPAC pAkt panel in Fig 5B when mirrored vertically.
- The MCF-7 β-actin panel in Fig 5A appears similar to the MCF-7 β-actin panel in Fig 5B.
- The HPAC β-actin panel in Fig 5A appears similar to the HPAC β-actin panel in Fig 5B.
- The HPAC Vimentin panel in Fig 5A appears similar to the HPAC HER1 panel in Fig 5B when mirrored vertically.
- The AspC-1 HER1 panel in Fig 5B appears similar to the AspC-1 pAKT panel in Fig 5B when exposure is adjusted.

The authors did not respond to queries about the experiments in Figs 2–5.

In light of the unresolved concerns discussed above, the *PLOS ONE* Editors retract this article.

All authors either did not respond directly or could not be reached.
